# Characterization of Multidrug-Resistant *Trueperella* (*Arcanobacterium*) *pyogenes* Isolates from Vertebral Osteomyelitis in Slaughtered Pigs

**DOI:** 10.3390/ani15202970

**Published:** 2025-10-14

**Authors:** In-Haeng Lee, Gun Lee, Hyeon Jeong Moon, Dae-Young Kim, Jong-Woog Choi, Yeong-Bin Baek, Sang-Ik Park, Dae-Sung Yoo, Jun Bong Lee, Bock-Gie Jung, Kwang-Jun Lee, Jun-Gyu Park

**Affiliations:** 1 Health and Environment Research Institute of Gwangju, Gwangju 61954, Republic of Korea; slayer19@korea.kr (I.-H.L.); daeyoung.keem@gmail.com (D.-Y.K.); lovnat@korea.kr (J.-W.C.); 2 Department of Veterinary Pathology, College of Veterinary Medicine and BK21 FOUR Program, Chonnam National University, Gwangju 61186, Republic of Korea; udlrjs77@naver.com (G.L.); dals203@naver.com (H.J.M.); sipark@jnu.ac.kr (S.-I.P.); 3 Department of Veterinary Pathology, College of Veterinary Medicine, Chonnam National University, Gwangju 61186, Republic of Korea; ybbaek@jnu.ac.kr; 4 Department of Veterinary Public Health, College of Veterinary Medicine, Chonnam National University, Gwangju 61186, Republic of Korea; shanuar@jnu.ac.kr; 5 Department of Food and Environmental Hygiene, College of Veterinary Medicine, Chonnam National University, Gwangju 61186, Republic of Korea; jblee12@jnu.ac.kr; 6 Department of Veterinary Microbiology, College of Veterinary Medicine, Chonnam National University, Gwangju 61186, Republic of Korea; bjung@jnu.ac.kr; 7 Division of Zoonotic and Vector-Borne Disease Research, Center for Infectious Diseases Research, National Institute of Health, Cheongju 28159, Republic of Korea; 8 Department of Veterinary Zoonotic Diseases, College of Veterinary Medicine, Chonnam National University, Gwangju 61186, Republic of Korea

**Keywords:** *Trueperella pyogenes*, slaughterhouse epidemiology, vertebral osteomyelitis, antimicrobial resistance, virulence factors

## Abstract

**Simple Summary:**

Slaughterhouses offer a valuable opportunity to detect hidden animal diseases that may not be observed during life. In this study, we examined fully condemned pig carcasses with spinal abscesses and identified *Trueperella pyogenes* as a key pathogen. By analyzing samples collected postmortem, we uncovered high levels of antibiotic resistance and evidence of potential virulence, providing critical insights into both animal health and food safety. This research demonstrates how slaughterhouse monitoring can support the early detection of emerging pathogens, track antimicrobial resistance, and guide farm-level interventions. It highlights the epidemiological value of routine meat inspection data for improving animal production systems and protecting public health.

**Abstract:**

Slaughterhouses serve as critical surveillance hubs for identifying subclinical and economically important diseases in food-producing animals. *Trueperella (Arcanobacterium) pyogenes*, an opportunistic pathogen commonly found on the mucous membranes of livestock, is associated with mastitis, abortion, and suppurative infections such as abscesses. In this study, we investigated 30 pig carcasses fully condemned due to vertebral osteomyelitis (VO) at two slaughterhouses in Gwangju, Republic of Korea, between November 2023 and May 2024. From abscess lesions, 11 *T. pyogenes* strains were isolated and characterized morphologically, biochemically, and genetically. The hemolytic exotoxin pyolysin (*plo* gene), a major virulence factor, was detected in five isolates (45.46%). Phylogenetic analysis of partial 16S rDNA sequences confirmed close clustering with known *T. pyogenes* reference strains. All 11 isolates exhibited multidrug resistance, showing resistance to 8–14 antimicrobial agents per strain. Complete resistance (11/11, 100%) was observed against amikacin (AMI), nalidixic acid (NAL), chloramphenicol (CHL), florfenicol (FFN), and trimethoprim/sulfamethoxazole (SXT). High resistance rates were also detected for erythromycin (ERY) and clindamycin (CLI) (10/11, 90.9%), ceftazidime (TAZ), ceftriaxone (AXO), ciprofloxacin (CIP) (7/11, 63.6%), and tetracycline (TET) and streptomycin (STR) (5/11, 45.5%), while gentamicin (GEN) resistance was found in three isolates (27.3%). In contrast, none of the isolates showed resistance to ampicillin, cefoxitin, or cefotaxime. These findings underscore the epidemiological value of abattoir-based monitoring in detecting emerging pathogens and tracking antimicrobial resistance. The results provide important baseline data to inform disease control strategies, guide antimicrobial stewardship, and support One Health approaches, including the development of preventive measures such as vaccines.

## 1. Introduction

*Trueperella pyogenes* is a Gram-positive, non-motile, non-capsulated, non-spore-forming facultatively anaerobic short rod-shaped bacterium either singly, in pairs, or in clusters [1,2]. Initially classified as *Bacillus pyogenes*, this organism has undergone several taxonomic reclassifications from *Corynebacterium pyogenes* to *Actinomyces pyogenes, Arcanobacterium pyogenes*, and finally, *T. pyogenes* [3]. Although its growth requirements are not complex, it requires media enriched with blood or serum for successful culture [1]. Early identification of *T. pyogenes* relies on its cell morphology, colony characteristics (such as β-hemolysis on blood agar), and a negative catalase test; subsequent biochemical testing including CAMP test, urease activity, nitrate reduction, oxidase test, gelatin and esculin hydrolysis, and fermentation of various carbohydrates (e.g., glucose, lactose, mannitol, maltose, sucrose, and xylose) aids in species confirmation [4]. As an opportunistic pathogen, this bacterium commonly colonizes the skin and mucosal surfaces of domestic animals like cattle, swine, sheep, goats, and chicken where it can cause infections such as mastitis, wound infections, pneumonia, and liver abscesses [1,5]. Infections in humans are rare and tend to occur sporadically, primarily in immunocompromised individuals with exposure to farm animals [1,2]. Exposure to this pathogen possibly induces vertebral osteomyelitis (VO), secondary to tail-biting or any wounds causing entry of bacterial pathogens [6], sepsis [7], endocarditis [3,8], pneumonia [9], and skin ulcers [10]. These various clinical manifestations and pathogenicity are known to be related with various virulence factors including pyolysin (*plo*), fimbrial proteins (*fimA*, *fimC*, *fimE*, *fimG*), neuraminidases (*nanH*, *nanP*), collagen-binding protein (*cbpA*), and superoxide dismutase (*sodA*) [2,11]. Among them, the *plo* gene encodes a cholesterol-dependent cytolysin that disrupts host cell membranes and is regarded as a key virulence determinant [12,13].

To date, no effective vaccine is available to prevent *T. pyogenes* infections [2]. For this reason, antimicrobial therapy remains the only available option for the prevention and treatment of VO caused by *T. pyogenes*. Commonly used antimicrobial agents include aminoglycosides, β-lactams, tetracyclines, macrolides, and fluoroquinolones [2]. However, the misuse and overuse of antibiotics in conventional pig farming systems have led to the emergence of antibiotic-resistant strains, thereby compromising the effectiveness of antimicrobial treatments [14]. Numerous studies have investigated the antimicrobial resistance (AMR) profiles of *T. pyogenes* across different countries, time periods, and animal species. Reported resistance rates vary, with tetracycline resistance ranging from 53.12% to 81.65%, streptomycin resistance from 32.6% to 72.48%, and erythromycin (ERY) resistance up to 21.10%. Lower resistance rates have been observed for ciprofloxacin (CIP) (around 12.5%), kanamycin (approximately 11.6%), and enrofloxacin (up to 21.87%). In contrast, all isolates in these studies remained susceptible to gentamicin (GEN), penicillin, and cephalothin [2,14,15,16].

In this study, we examined 30 pig carcasses fully condemned due to VO at two slaughterhouses in Gwangju, Republic of Korea, between November 2023 and May 2024. Using a combination of bacterial culture, molecular identification, PCR-based toxin gene detection, and antimicrobial susceptibility testing, we characterized 11 *T. pyogenes* isolates. This study was conducted to better understand the etiological role of *T. pyogenes* in swine VO, a condition that leads to significant carcass condemnation and economic loss. Our aim was to identify the pathogen’s AMR patterns and virulence profiles to support early detection and control strategies at the slaughterhouse level. In addition, we sought to assess its potential as a zoonotic pathogen, given the risk of transmission to humans and its implications for public health.

## 2. Materials and Methods

### 2.1. Bacteria Isolation and Culture Conditions

In 2024, a total of 470,414 pigs were slaughtered in the Gwangju area, among which 0.033% of carcasses were condemned. As part of our monitoring between November 2023 and May 2024, 235,430 pigs were examined, and among them, 30 pigs were diagnosed with vertebral osteomyelitis (VO). The affected pigs included 14 females and 16 castrated males. Of these, 29 pigs had been raised in Jeollanam-do, while 1 was raised in Gyeongsangbuk-do, a province located in the southeastern region of the country. Swab samples were collected directly from the abscesses after recording the sex and carcass weight of each animal (Table 1). The abscesses were in the vertebrae, including the cervical, thoracic, and/or lumbar regions. As the lesions were identified postmortem, no clinical signs were observed prior to slaughter.

### 2.2. Virulence Factor Analysis

To confirm the presence of toxins in the isolated *T. pyogenes* strains, PCR analysis was performed. Briefly, a single colony of the pure isolate was selected and mixed with 1 mL of sterile PBS, followed by vortex. The mixture was then heat-treated at 100 °C for 15 min and centrifuged at 12,000 rpm for 15 min. The supernatant was used as the DNA template. PCR was conducted with the primer of F: 5′-GGC CCG AAT GTC ACC GC-3′ (positions 823–839) and R: 5′-AAC TCC GCC TCT AGC GC-3′ (positions 1092–1076), with an annealing temperature 55 °C, yielding a product size of 270 bp to detect the hemolytic exotoxin pyolysin (*plo* gene), a major virulence factor of *T. pyogenes* [17]. After PCR, the products were analyzed using 1.5% agarose gel electrophoresis at 100 V for 30 min using Bio-Rad Mini-Sub Cell GT System (Bio-Rad, Hercules, CA, USA), and the presence of bands was used to determine positive results [18].

### 2.3. Antimicrobial Susceptibility Test

The antimicrobial susceptibility of *T. pyogenes* isolates was assessed by determining the minimum inhibitory concentrations (MICs) using the broth microdilution method with Sensititre panel (TREK Diagnostic System, Cleveland, OH, USA). Briefly, *T. pyogenes* colonies grown on blood agar plate (Synergy inovation, Seongnam, Republic of Korea) were suspended in 5 mL of sterile saline to obtain a McFarland standard of 0.5. Bacterial cell suspensions were diluted with 11 mL of Tryptic soy broth (BD Difco, Sparks, MD, USA) supplemented with 5% lysed horse blood (MBcell, Seoul, Republic of Korea) and dispensed onto the Sensititre panel. The panels were incubated at 37 °C with anaerobic condition for 96 h, and susceptibility was interpreted according to the criteria used in this study.

Using the Sensititre panel, 17 antibiotics including ampicillin (AMP, 1–64 µg/mL), cefoxitin (FOX, 4–8 µg/mL), ceftazidime (TAZ, 1–16 µg/mL), cefotaxime (FOT, 1–32 µg/mL), ceftriaxone (AXO, 0.25–64 µg/mL), tetracycline (TET, 2–128 µg/mL), GEN (1–64 µg/mL), streptomycin (STR, 2–128 µg/mL), amikacin (AMI, 4–64 µg/mL), CIP (0.03–16 µg/mL), nalidixic acid (NAL, 2–128 µg/mL), colistin (COL, 2–16 µg/mL), chloramphenicol (CHL, 2–32 µg/mL), florfenicol (FFN, 0.03–64 µg/mL), azithromycin (AZI, 2–32 µg/mL), trimethoprim/sulfamethoxazole (SXT, 0.12/2.38–4/76 µg/mL), clindamycin (CLI, 0.03–16 µg/mL), and ERY (0.03–64 µg/mL) were tested. The MIC breakpoint used to classify isolates as susceptible or resistant to each antibiotic was identified using previous reports, TET, GEN, STR [19], TAZ, FOT, AMI, CIP, CHL, FFN, AZI, CLI antibiotics [20]; CLSI document M100 (34th edition, 2024) for AXO, FOX, NAL antibiotics; CLSI document VET06 (CLSI, 2017) for AMP, SXT and ERY. *Escherichia coli* ATCC 25922 and *Staphylococcus aureus* ATCC 25923 were used as internal quality control strains for antimicrobial susceptibility test. Based on the standards outlined in Table 2, isolates were classified as susceptible (S) or resistant (R) [19,20,21,22].

### 2.4. Analysis of 16S rRNA Gene Partial Sequences

The phylogenetic relationship of *T. pyogenes* isolates was analyzed in comparison to reference strains by targeting the species-specific region of the 16S rRNA intergenic spacer region (ISR). The 16S rRNA gene was partially amplified using primers F8 (5′-GAGTTTGATCCTGGCTCAG-3′) and 1492R (5′-GGACTACCAGGGTATCTAAT-3′), as previously described [23,24]. The amplified fragments were purified with the Illustra GFX™ PCR DNA and Gel Band Purification Kit (GE Healthcare, Chicago, IL, USA) and the amplified partial 16S rRNA gene was sequenced using an Applied Biosystems automated sequencer (ABI 3730XL) at Macrogen Co., Ltd. (Seoul, Republic of Korea). The resulting sequences were analyzed using BLAST version 2.13.0+ against the GenBank nucleotide non-redundant database. The 16S rRNA sequences generated in this study have been submitted to NCBI GenBank. The 16S rRNA gene sequences were aligned and used to construct a phylogenetic tree. The tree was generated using the neighbor-joining (NJ), kimura 2-parameter model in MEGA (version 12), with 1000 bootstrap replications performed as previously described [25].

### 2.5. Statistical Analysis

Welch’s *t*-test was conducted using Prism software version 10.4.1 (GraphPad, San Diego, CA, USA) to compare the mean body weights between pigs with VO and healthy pigs. Statistical significance was defined as follows: *p* < 0.05 (*), *p* < 0.01 (**), and *p* < 0.001 (***).

## 3. Results

### 3.1. Characteristics of Vertebral Osteomyelitis Cases

**Figure 1:**
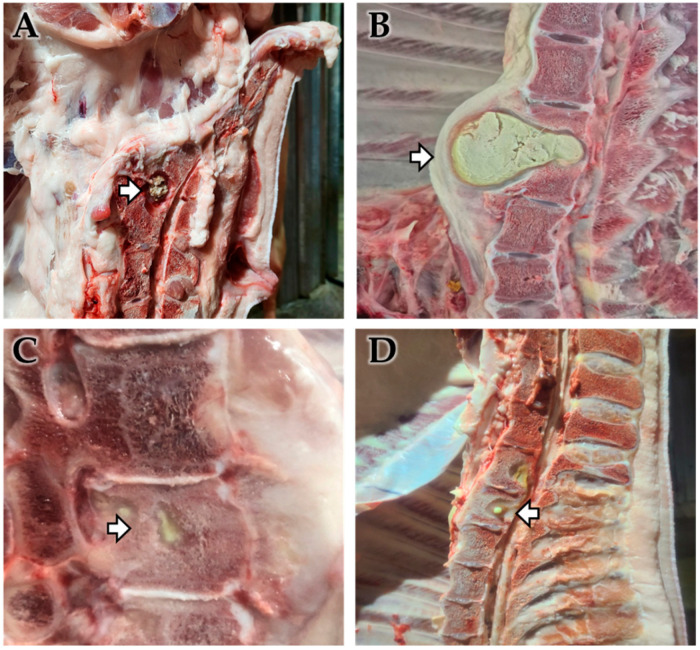
Gross observation of vertebral osteomyelitis (VO) cases. Gross pathological findings of a VO in pigs. Abscesses (arrow) were found from the intervertebral disks (**A**,**B**) or inside of thoracic vertebrae (**C**,**D**). Each lesion revealed extensive suppurative inflammation and tissue destruction in the thoracic vertebral bone and spinal column, with lesions filled with necrotic debris and infiltration into surrounding tissues, indicating a chronic suppurative process associated with bacterial infection.

**Figure 2:**
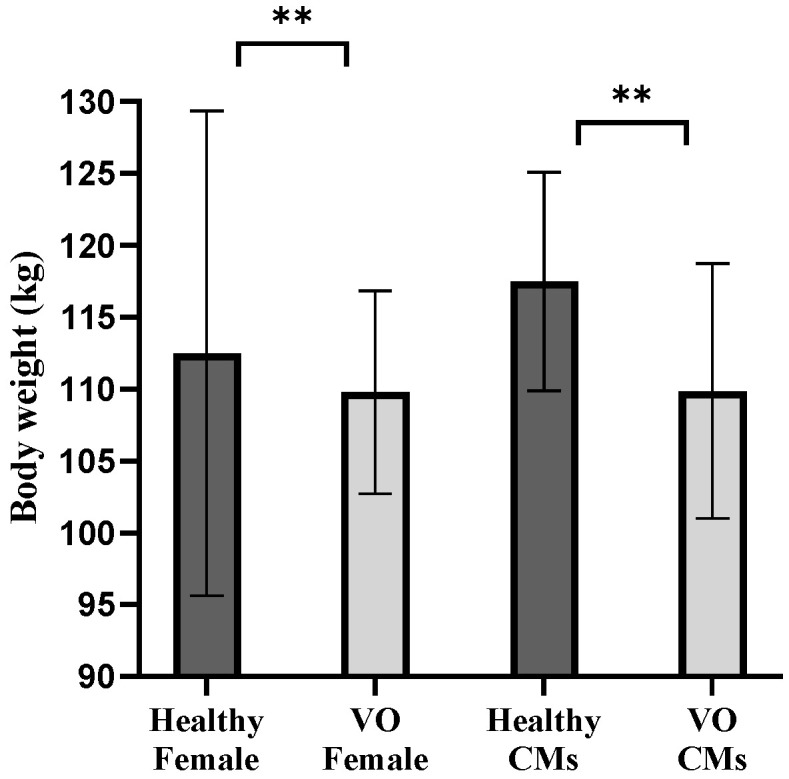
Weight differences between vertebral osteomyelitis (VO) and healthy pigs. The mean body weights (kg) of healthy females, VO females, healthy castrated males (CMs), and VO-CMs are shown as bar graphs. Statistical analysis was performed using Welch’s *t*-test. A statistically significant difference was observed between the healthy-CMs and VO-CMs groups (*p* < 0.01), indicated by ** and between the healthy-Female and VO-Female groups (*p* < 0.01), also indicated by ** Significance levels are represented as follows: *p* < 0.05 (*), *p* < 0.01 (**), and *p* < 0.001 (***).

Bacterial culture revealed that *T. pyogenes* was isolated from 11 of 30 VO samples (isolation rate: 36.7%). Among the 11 samples where *T. pyogenes* was identified, 8 were from females (out of 14 total female samples; 57.1%) and 3 were from castrated males (out of 16 total castrated male samples; 18.8%) (Figure 1, Table 1). The average body weight of pigs with VO was 4.8% lower in females (107.07 ± 5.51 kg, *n* = 14) compared to healthy female controls (112.48 ± 16.844 kg, *n* = 100,356), and 5.6% lower in castrated males (110.87 ± 8.54 kg, *n* = 16) compared to healthy castrated-male controls (117.47 ± 7.60 kg, *n* = 135,074) at the time of slaughter (Figure 2, Table 1).

### 3.2. Bacteria Screening and Isolation

A total of 30 spinal abscess samples were cultured on MacConkey agar, Baird-Parker agar, XLD agar, and EMB agar. All 11 *T. pyogenes*-positive isolates formed pinpoint, shiny, β-hemolytic colonies on blood agar. No colony growth was observed on MacConkey, Baird-Parker, or XLD agar. Although colonies were present on EMB agar, none exhibited the characteristic metallic green sheen, which is typically associated with *E. coli* rather than *T. pyogenes*.

### 3.3. Virulence Related Gene Examination

To investigate bacterial pathogenicity in the 11 *T. pyogenes* isolates, the *plo* toxin gene, a key virulence factor, was examined using PCR analysis. As a result, 5 out of 11 *T. pyogenes* strains (IH2311HS_GJ01, IH2311.1MA_GJ02, IH2311.2MA_GJ03, IH2312KC_GJ05, and IH2312GM_GJ06) showed positive for *plo* gene (Table 3).

### 3.4. Antimicrobial Susceptibility Test

**Figure 3:**
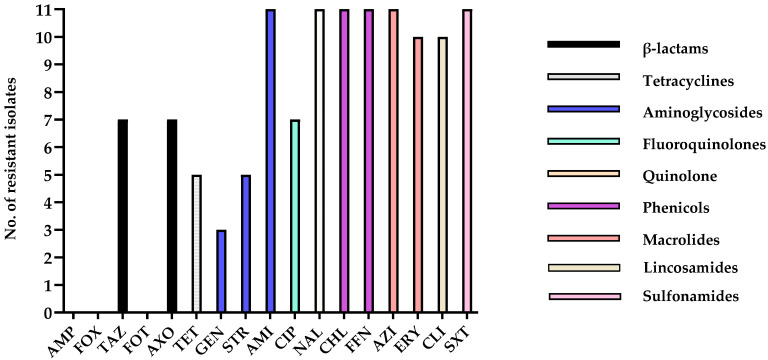
Number of antimicrobial-resistant isolates. This graph shows the number of resistant isolates for each antimicrobial agent tested. The x-axis represents the antimicrobial agents, and the y-axis represents the number of isolates that showed resistance to a specific antimicrobial agent based on the CLSI (or other relevant standards) breakpoints.

Through the antimicrobial susceptibility test, all 11 isolates exhibited multidrug resistance, with resistance observed to between 8 and 14 antimicrobial agents per strain (Table 4). When grouped by antibiotic classes, most β-lactams exhibited susceptibility across isolates, with the exception of TAZ, to which 7 of 11 isolates were resistant. In the tetracycline class, 5 out of 11 isolates displayed resistance. Among aminoglycosides, GEN resistance was limited to three isolates, while STR resistance was identified in 5 of 11 isolates, and all isolates were resistant to AMI. In contrast, high resistance rates were observed across other antibiotic classes, with all isolates resistant to fluoroquinolones (CIP), quinolones (NAL), phenicols (CHL, FFN), macrolides (AZI, ERY), Sulfonamides (SXT), and lincosamides (CLI) (Table 4 and Figure 3).

### 3.5. Analysis of 16S rRNA Gene Partial Sequences

**Figure 4:**
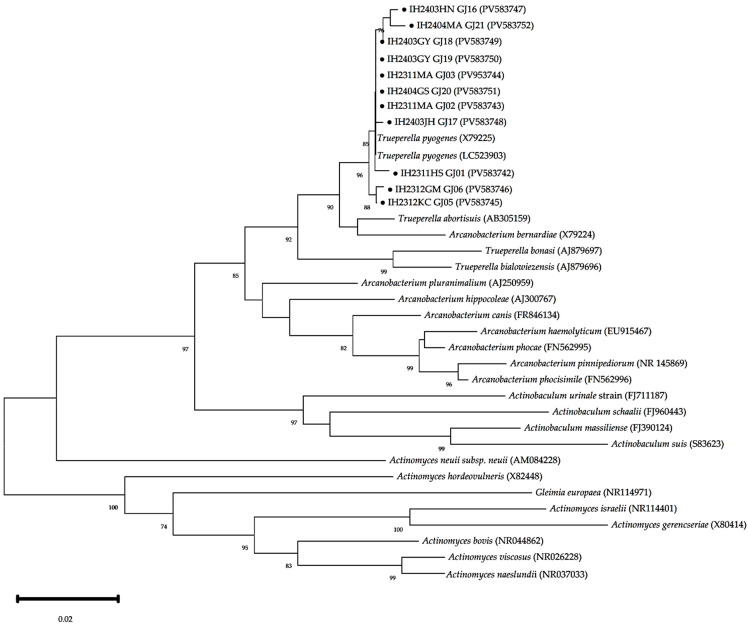
Phylogenic analysis of *T. pyogenes* based on 16S rRNA gene sequences. The phylogenetic tree was constructed using the neighbor-joining method with the Kimura 2-parameter model in MEGA version 12, based on partial 16S rRNA gene sequences. Black circles (●) indicate isolates used in this study. These isolates clustered closely with reference strains of *T. pyogenes* (accession No. NCTC5225, LC259203), confirming their identification. Bootstrap values (≥70%) from 1000 replicates are shown at the corresponding nodes. The scale bar represents 0.02 nucleotide substitutions per site.

The phylogenetic analysis based on 16S rRNA gene sequences showed that isolates IH2311HS_GJ01 (accession No. PV583742), IH2311.1MA_GJ02 (accession No. PV583743), IH2311.2MA (accession No. PV583744), IH2312KC_GJ05 (accession No. PV583745), IH2312GM_GJ06 (accession No. PV583746), IH2403HN_GJ16 (accession No. PV583747), IH2403JH_GJ17 (accession No. PV583748), IH2403YG_GJ18 (accession No. PV583749), IH2403GY_GJ19 (accession No. PV583750), IH2404GS_GJ20 (accession No. PV583751), and IH2404MA_GJ21 (accession No. PV583752) clustered closely with reference strains of *T. pyogenes* (e.g., X79225 and LC523903) in the phylogenetic tree (Figure 4). The nucleotide sequence homology varied from 98.27% to 99.66% for LC523903 and from 98.52% to 99.59% for X79225. Sequence analysis that the isolates in this study belong to *T. pyogenes*, as they showed lower homology (97.05–98.07%) with other *Trueperella* species, including *T. abortisuis*, the closest known relative (Figure 4).

## 4. Discussion

*T. pyogenes* is widely recognized as an opportunistic bacterium that colonizes the skin and mucosal surfaces of the upper respiratory, gastrointestinal, and urogenital tracts of animals [1,2,26]. Although it has been known for decades, the mechanisms underlying its pathogenicity, transmission dynamics, and environmental reservoirs remain only partially understood. The organism is implicated in various suppurative infections such as mastitis, metritis, pneumonia, and abscess formation that result in significant economic losses in livestock production systems.

In 2024, a total of 470,414 pigs were slaughtered in the Gwangju area, among which 154 (0.033%) carcasses were condemned. This result is consistent to the previous study conducted in Korea that the occurrence of VO in slaughtered pigs ranged from 0.03% to 0.07% [26]. Notably, 68 cases (44.2%) were attributed to VO cases. The next most common causes of carcass condemnation were rectal stricture (33.7%), abnormal odor (12.9%), and peritonitis (5.8%), in decreasing order of frequency (Unpublished data). Among the pathogens implicated in VO, *T. pyogenes* is particularly notable for its ability to cause deep tissue infections in swine and ruminants [10,26,27,28].

This study reinforces the value of slaughterhouses as sentinel sites for animal disease surveillance. Postmortem inspection provides a unique opportunity to detect chronic, subclinical, or localized infections that may not manifest overt clinical signs on farms. As such, slaughterhouse-based monitoring not only reflects the health status of regional livestock populations but also offers a cost-effective strategy for detecting zoonotic pathogens and tracking AMR trends [29,30]. These data are critical for both veterinary public health and food safety systems, aligning with global One Health priorities.

AMR is an escalating global health concern, and food-producing animals are increasingly recognized as reservoirs for resistant bacteria [29,30,31]. Despite the widespread presence of *T. pyogenes*, no commercial vaccine is currently available, making antimicrobial treatment the primary therapeutic strategy [1,11,18]. However, the efficacy of such treatments is increasingly jeopardized by the emergence of resistant strains, often driven by the routine and sometimes excessive use of antibiotics in agriculture [32,33,34,35,36,37,38,39]. Previous studies have reported variable antimicrobial resistance rates of *T. pyogenes* depending on the host species and clinical conditions. For instance, resistance to streptomycin ranged from 32.6% to 81.25%, tetracycline from 32.6% to 53.12%, and erythromycin from 28.12% to 85.20%, with generally high resistance observed in isolates from cows with mastitis or metritis. In contrast, most isolates remained susceptible to penicillin, gentamicin, and cephalexin in certain cases, while resistance to ampicillin, ciprofloxacin, and enrofloxacin was considerably higher in bovine and caprine isolates. Notably, the current study revealed high resistance rates to several antibiotics including amikacin, florfenicol, chloramphenicol, azithromycin, clindamycin, and trimethoprim-sulfamethoxazole in swine-derived isolates, suggesting an emerging multidrug-resistant profile (Table 5) [2,11,40,41]. In the current study, all 11 *T. pyogenes* isolates from VO cases exhibited AMR, with resistance patterns ranging from 8 to 14 antibiotics per isolate. Notably, resistance was observed against antibiotic classes commonly used in swine farms, including tetracyclines (TET), phenicols (CHL, FFN), aminoglycosides (STR, AMI, kanamycin), and macrolides (tylosin, ERY). Resistance to these classes is particularly concerning, as they represent frontline drugs frequently employed for both therapeutic and prophylactic purposes in pig production. The widespread resistance to tetracyclines and macrolides may reflect long-term and repeated use in feed additives, while resistance to aminoglycosides and phenicols indicates the limited efficacy of these traditionally relied-upon treatments. These results not only indicate the rise in multidrug-resistant strains but also suggest that commonly used antibiotics may no longer be effective for treating bacterial infections in livestock. This underscores the urgent need for continuous AMR surveillance, stricter regulation of antimicrobial use, and the promotion of prudent veterinary-guided antibiotic selection to preserve the effectiveness of existing drugs and ensure sustainable livestock production.

Zoonotic risk associated with *T. pyogenes* is significant, as this bacterium serves as a common commensal in various animal species, acting as a reservoir for AMR genes. Importantly, resistant strains may cross species barriers and be transmitted to humans, where they pose a therapeutic challenge [3,42,43]. Although human cases of *T. pyogenes* infections are relatively rare, they have been documented in clinical settings, including severe manifestations such as sepsis [7,44], endocarditis [3,8,45], ophthalmic and central nervous system infection [46], soft tissue infection [10,47], and skin ulcers [1,3,8,18,46], suggesting its zoonotic potential and occurrence across different regions of the world. In animals, it has been associated with reproductive, respiratory, and dermatological conditions, further amplifying its impact on productivity and animal welfare [8,48]. Therefore, continuous monitoring and a deeper understanding of its transmission pathways are essential to safeguard both human and animal health.

The diverse clinical manifestations associated with *T. pyogenes* infections are mediated by a range of virulence factors, including *plo*, neuraminidases (nanP, nanH), and fimbrial proteins (*fimA*, *fimC*, *fimE*). A recent study from China reported that all *T. pyogenes* isolates (total 86 strains) obtained from goats and sheep harbored the *plo* gene, regardless of the clinical manifestation, which included pneumonia, subcutaneous abscesses, mastitis, ulcerated skin, and metritis [11]. In contrast, only 45.5% of the isolates in the present study, which were collected from pigs with VO, were positive for the *plo* gene and no clear association was observed between the presence of the *plo* gene and antimicrobial susceptibility profiles (Table 3 and Table 4). This discrepancy suggests that the presence of *plo* alone may not be sufficient to explain specific tissue tropism, such as VO, and that the genotypic profiles of virulence factors vary across host species. However, despite collecting VO cases for one year, only 11 isolates were available for analysis, which limits the representativeness of the findings. Furthermore, the exclusive focus on the *plo* gene does not capture the broader virulence gene repertoire, and the association between virulence markers and clinical manifestations remains unclear. Vertebral infections in pigs are often attributed to ascending infections from tail wounds caused by fighting between animals, suggesting that both the infection route and specific gene expression patterns may influence disease localization. To address these gaps, future studies should include larger sample sizes, investigate multiple virulence factors beyond *plo*, and employ functional approaches such as in vivo models to clarify the pathogenic mechanisms and better define the clinical relevance of virulence gene expression in VO.

## 5. Conclusions

This study identified *T*. *pyogenes* as the primary pathogen responsible for vertebral abscesses that led to carcass condemnation in pigs at slaughter. The isolates demonstrated multidrug resistance, indicating the ongoing antimicrobial pressure within livestock production environments. These results reinforce the importance of slaughterhouses as practical surveillance points, where postmortem examination allows for the detection of both clinically apparent and subclinical infections. Such surveillance efforts play a key role in tracking antimicrobial resistance trends and understanding pathogen dynamics in food animals, providing valuable insights for animal health management and food safety monitoring.

## Figures and Tables

**Table 1:** Information of pigs used in this study.

Sample	Weight (kg)	Relative Body Weight to Control Pigs (%)	Sex	Collection Date
H2311HS_GJ01	103 kg	91.57%	Female	16 November 2023
IH2311.1MA_GJ02	113 kg	96.2%	Castrated	16 November 2023
IH2311.2MA_GJ03	112 kg	99.58%	Female	23 November 2023
IH2312KC_GJ05	100 kg	88.91%	Female	12 December 2023
IH2312GM_GJ06	82 kg	69.81%	Castrated	12 December 2023
IH2403HN_GJ16	114 kg	101.35%	Female	20 March 2024
IH2403JH_GJ17	111 kg	98.69%	Female	21 March 2024
IH2403YG_GJ18	115 kg	102.24%	Female	22 March 2024
IH2403GY_GJ19	115 kg	102.24%	Female	26 March 2024
IH2404GS_GJ20	104 kg	92.47%	Female	1 April 2024
IH2404MA_GJ21	119 kg	101.3%	Castrated	4 April 2024

**Table 2:** Antimicrobial agents used in this study and their minimal inhibitory concentration breakpoint.

Antibiotic Class	Antibiotic Name (Abbreviation)	Minimal Inhibitory Concentration Breakpoint (µg/mL)	
		Susceptible (S)	Resistant (R)
β-lactams	Ampicillin (AMP)	≤1	≥2
	Cefoxitin (FOX)	≤4	≥8
	Ceftazidime (TAZ)	≤4	≥8
	Cefotaxime (FOT)	≤8	≥16
	Ceftriaxone (AXO)	≤0.5	≥1
Tetracyclines	Tetracycline (TET)	≤2	≥4
Aminoglycosides	Gentamicin (GEN)	≤8	≥16
	Streptomycin (STR)	≤4	≥8
	Amikacin (AMI)	≤8	≥16
Fluoroquinolones	Ciprofloxacin (CIP)	≤2	≥4
Quinolone	Nalidixic acid (NAL)	≤16	≥32
Phenicols	Chloramphenicol (CHL)	≤0.5	≥1
	Florfenicol (FFN)	≤0.5	≥1
Macrolides	Azithromycin (AZI)	≤4	≥8
	Erythromycin (ERY)	≤4	≥8
Sulfonamides	Trimethoprim/Sulfamethoxazole (SXT)	≤0.12	≥0.25
Lincosamides	Clindamycin (CLI)	≤4	≥8

**Table 3:** Detection of *plo* toxin gene from *T. pyogenes*-positive colonies.

Sample	*plo* Gene PCR Result
H2311HS_GJ01	+
IH2311.1MA_GJ02	+
IH2311.2MA_GJ03	+
IH2312KC_GJ05	+
IH2312GM_GJ06	+
IH2403HN_GJ16	−
IH2403JH_GJ17	−
IH2403YG_GJ18	−
IH2403GY_GJ19	−
IH2404GS_GJ20	−
IH2404MA_GJ21	−

**Table 4:** Antimicrobial susceptibility profiles of *T. pyogenes* isolates based on Sensititre plate assay.

	AMP	FOX	TAZ	FOT	AXO	TET	GEN	STR	AMI	CIP	NAL	CHL	FFN	AZI	ERY	CLI	SXT	No. of Resistant Antimicrobial Agents
Sample	Minimum Inhibitory Concentrations (MICs, µg/mL)/ Susceptibility (S: Susceptible, R: Resistant)																	
IH2311HS_GJ01	<1/ S	<4/ S	4/ S	<1/ S	2/ R	64/ R	>64/ R	>128/ R	64/ R	4/ R	>128/ R	32/ R	8/ R	>32/ R	>64/ R	>16/ R	0.5/ R	13
IH2311.1MA_GJ02	<1/ S	<4/ S	4/ S	<1/ S	2/ R	<2/ S	4/ S	4/ S	16/ R	2/ S	>128/ R	8/ R	2/ R	>32/ R	>64/ R	>16/ R	1/ R	9
IH2311.2MA_GJ03	<1/ S	<4/ S	8/ R	<1/ S	2/ R	64/ R	>64/ R	>128/ R	64/ R	4/ R	>128/ R	8/ R	2/ R	>32/ R	>64/ R	>16/ R	0.25/ R	14
IH2312KC_GJ05	<1/ S	<4/ S	8/ R	<1/ S	2/ R	<2/ S	8/ S	4/ S	16/ R	1/ S	>128/R	8/ R	2/ R	>32/ R	>64/ R	>16/ R	2/ R	10
IH2312GM_GJ06	<1/ S	<4/ S	8/ R	<1/ S	1/ R	<2/ S	4/ S	4/ S	16/ R	2/ S	>128/R	8/ R	1/ R	>32/ R	0.06/ S	0.12/ S	0.5/ R	8
IH2403HN_GJ16	<1/ S	<4/ S	8/ R	<1/ S	1/ R	<2/ S	4/ S	4/ S	16/ R	4/ R	>128/ R	8/ R	2/ R	>32/ R	>64/ R	>16/ R	1/ R	11
IH2403JH_GJ17	<1/ S	<4/ S	8/ R	<1/ S	1/ R	<2/ S	4/ S	4/ S	16/ R	4/ R	>128/ R	8/ R	2/ R	>32/ R	>64/ R	>16/ R	1/ R	11
H2403YG_GJ18	<1/ S	<4/ S	<1/ S	<1/ S	1/ S	64/ R	8/ S	>128/ R	32/ R	4/ R	>128/ R	8/ R	2/ R	>32/ R	>64/ R	>16/ R	0.5/ R	11
IH2403GY_GJ19	<1/ S	<4/ S	4/ S	<1/ S	0.25/ S	64/ R	8/ S	>128/ R	32/ R	4/ R	>128/ R	8/ R	2/ R	>32/ R	>64/ R	>16/ R	0.5/ R	11
IH2404GS_GJ20	<1/ S	<4/ S	16/ R	<1/ S	0.25/ S	<2/ S	<4/ S	4/ S	16/ R	4/ R	>128/ R	8/ R	2/ R	>32/ R	>64/ R	>16/ R	1/ R	10
IH2404MA_GJ21	<1/ S	<4/ S	16/ R	<1/ S	0.5/ S	32/ R	>64/ R	>128/ R	64/ R	2/ S	>128/ R	32/ R	16/ R	>32/ R	>64/ R	>16/ R	0.25/ R	12

**Table 5:** Comparison of antimicrobial resistance rates of *T*. *pyogenes* isolates among different animal species.

Antibiotic Class	Antibiotic	Pig (This Study)	Pig (Pneumonia) [40]	Cow (Mastitis) [41]	Cow (Metritis) [41]	Sheep/ Goat [2,11]
β-lactams	Amoxicillin	–	0%	87.50%	100%	–
	Amoxicillin/ Clavulanic acid	–	–	–	–	38.40%
	AMP	0%	–	90.62%	100%	–
	Cefalexin	–	–	84.37%	97.56%	–
	Cefazolin	–	7.40%	–	–	–
	FOT	0%	–	–	–	–
	FOX	0%	–	–	–	–
	TAZ	63.60%	–	–	–	–
	AXO	63.60%	–	–	–	–
	Penicillin	–	0%	100%	97.56%	0.00–5.80%
	AXO	63.60%	–	–	–	–
Tetracyclines	TET	45.50%	–	53.12%	48.78%	32.60–53.12%
Aminoglycosides	GEN	27.30%	77.80%	100%	97.56%	21.87
	STR	45.50%	–	81.25%	56.09%	32.60–56.25%
	AMI	100%	74.10%	–	–	–
Fluoroquinolones	CIP	63.60%	0%	53.12%	70.73%	–
	Enrofloxacin	–	0%	59.37%	73.17%	78.13
Quinolone	NAL	100%	–	–	–	–
Phenicols	FFN	100%	0%	–	–	–
	CHL	100%	–	–	–	14.00%
Macrolides	AZI	100%	85.20%	37.50%	46.34%	–
	Tylosin	–	–	62.50%	63.41%	–
	ERY	90.90%	85.20%	53.12%	39.02%	28.12–44.20%
	Clindamycin	90.90%	–	–	–	4.70%
Sulfonamides	SXT	100%	–	87.50%	70.73%	37.50%
	Sulfisoxazole	–	–	–	–	37.20%
Lincosamides	Lincomycin	–	–	43.75%	58.53%	–
Rifamycins	Rifampicin	–	–	59.37%	68.29%	–

## Data Availability

All data in this study are included in the article.
